# A comparison of target volumes drawn on arterial and venous phase scans during radiation therapy planning for patients with pancreatic cancer: the PANCRINJ study

**DOI:** 10.1186/s13014-024-02477-8

**Published:** 2024-07-15

**Authors:** Fabien Zaidi, Paul Calame, Cédric Chevalier, Julie Henriques, Dewi Vernerey, Lucine Vuitton, Bruno Heyd, Christophe Borg, Jihane Boustani

**Affiliations:** 1 Department of Radiotherapy, University of Bourgogne Franche-Comté, CHU Besançon, CHRU Besançon, Service de Radiothérapie, Hôpital Jean Minjoz, 3 Boulevard Alexandre Fleming, Besançon, 25030 France; 2 Department of Radiology, University of Bourgogne Franche-Comté, CHU Besançon, Besançon, 25030 France; 3 Methodology and Quality of Life Unit in Oncology, University Hospital of Besançon, Besançon, France; 4 Université de Franche-Comté, EFS, INSERM, UMR RIGHT, Besançon, F-25000 France; 5 Department of Gastroenteroly, University of Bourgogne Franche-Comté, CHU Besançon, Besançon, 25030 France; 6 Department of Digestive surgery, University of Bourgogne Franche-Comté, CHU Besançon, Besançon, 25030 France; 7 Department of Oncology, University of Bourgogne Franche-Comté, CHU Besançon, Besançon, 25030 France

**Keywords:** Pancreatic cancer, Radiation therapy, Arterial phase, Venous phase, Inter-observer variability

## Abstract

**Background:**

The planification of radiation therapy (RT) for pancreatic cancer (PC) requires a dosimetric computed tomography (CT) scan to define the gross tumor volume (GTV). The main objective of this study was to compare the inter-observer variability in RT planning between the arterial and the venous phases following intravenous contrast.

**Methods:**

PANCRINJ was a prospective monocentric study that included twenty patients with non-metastatic PC. Patients underwent a pre-therapeutic CT scan at the arterial and venous phases. The delineation of the GTV was performed by one radiologist (gold standard) and two senior radiation oncologists (operators). The primary objective was to compare the Jaccard conformity index (JCI) for the GTVs computed between the GS (gold standard) and the operators between the arterial and the venous phases with a Wilcoxon signed rank test for paired samples. The secondary endpoints were the geographical miss index (GMI), the kappa index, the intra-operator variability, and the dose-volume histograms between the arterial and venous phases.

**Results:**

The median JCI for the arterial and venous phases were 0.50 (range, 0.17–0.64) and 0.41 (range, 0.23–0.61) (*p* = 0.10) respectively. The median GS-GTV was statistically significantly smaller compared to the operators at the arterial (*p* < 0.0001) and venous phases (*p* < 0.001), respectively. The GMI were low with few tumors missed for all patients with a median GMI of 0.07 (range, 0-0.79) and 0.05 (range, 0-0.39) at the arterial and venous phases, respectively (*p* = 0.15). There was a moderate agreement between the radiation oncologists with a median kappa index of 0.52 (range 0.38–0.57) on the arterial phase, and 0.52 (range 0.36–0.57) on the venous phase (*p* = 0.08). The intra-observer variability for GTV delineation was lower at the venous phase than at the arterial phase for the two operators. There was no significant difference between the arterial and the venous phases regarding the dose-volume histogram for the operators.

**Conclusions:**

Our results showed inter- and intra-observer variability in delineating GTV for PC without significant differences between the arterial and the venous phases. The use of both phases should be encouraged. Our findings suggest the need to provide training for radiation oncologists in pancreatic imaging and to collaborate within a multidisciplinary team.

**Supplementary Information:**

The online version contains supplementary material available at 10.1186/s13014-024-02477-8.

## Background

The incidence of pancreatic cancer (PC) increased substantially over the years. In 2020, 495,773 new cases were diagnosed with 466,003 deaths worldwide [1]. In France, PC accounted for 11,456 deaths and 14,184 new cases in 2018 [2]. At diagnosis, 40–50% of the patients are metastatic, and 35–40% have unresectable PC which can be either borderline or locally advanced [3]. Only 15–20% of the patients have a localized tumor eligible to curative surgery. The role of radiation therapy (RT) in the management of PC is controversial. For locally advanced PC, the standard treatment is chemotherapy (CT) with FOLFIRINOX in fit patients [4]. RT can be discussed after induction CT in selected patients without progressive disease [5, 6]. In the LAP07 phase 3 trial, in patients controlled after four months of gemcitabine-based induction CT, locoregional progression was less frequent in those who received chemoradiation (CRT) compared to CT alone (32% vs. 46%, *p* = 0.03) [5]. The median time without treatment was significantly longer (6.1 vs. 3.7 months, *p* = 0.017), with no statistically significant difference in progression-free survival (9.9 vs. 8.4 months, *p* = 0.06) [5]. For borderline tumors, resection is considered after neoadjuvant CT [6]. Pre-operative CRT seems to improve the complete resection rate as well as the disease-free and locoregional failure-free survival [7, 8].

To plan RT, a dosimetric computed tomography (CT) scan is performed with or without intravenous (IV) contrast. Practices vary among centers since this CT scan can be done without IV contrast injection, or with IV contrast at the arterial or at the venous phases [7]. During the RT planning, the delineation of the gross tumor volume (GTV) is a critical step. However, the delineation of target volumes remains subjective, resulting in significant differences between observers. For instance, in the analysis of the pre-trial benchmark case for the selective CRT in advanced localized PC study SCALOP, the GTVs delineated by 25 radiation oncologists were compared to a reference GTV [8]. The median Jaccard conformity Index (JCI) was 0.57 (0.51–0.65) with a JCI of 1 representing total agreement. This inter-observer variability is a source of uncertainty which can have consequences on tumor control, survival, and normal tissue sparing [9, 10]. Having a greater contrast between the tumor and the normal pancreatic parenchyma could increase the accuracy of the delineation and reduce the heterogeneity in RT planning. It has been shown that the difference in contrast enhancement between the pancreatic gland and the tumor is maximal at the arterial phase [11, 12]. On the other hand, the portal venous phase improves the visualization of vasculature. Thus, we sought to compare the pancreatic tumor delineation on CT scans performed at the arterial and venous phases in patients with non-metastatic PC. To evaluate the inter-observer variability, the GTVs defined by two radiation oncologists (operators) were compared to those done by a radiologist specialized in pancreatic imaging (gold standard (GS)).

## Methods

### Patients

Between 11/12/2021 and 03/23/2023, 20 patients with pancreatic adenocarcinoma having a CT scan for the diagnosis of the disease or for an evaluation after treatment were prospectively included in a single French university hospital. All patients had a pancreatic biopsy to confirm the diagnosis. Other histologies including neuroendocrine tumors or non-invasive cancers were excluded. Patients with a pancreatic ductal adenocarcinoma (PDA) suitable for RT, such as resectable tumors, borderline resectable tumors, and locally advanced tumors were included. Patients with a metastatic disease were excluded. The patients’ clinical data were anonymized and could be used to help delineation. We used the National Comprehensive Cancer Network (NCCN) classification for resectable PDA defined as no vascular contact with the superior mesenteric artery (SMA), the coeliac axis (CA), and the common hepatic artery (CHA), and less than 180° contact with the superior mesenteric vein (SMV) and the portal vein (PV), with no evidence of vein distortion [13]. Borderline resectable PDA was defined as reconstructible involvement of the SMV or portal vein and less than 180° contact of the SMA and CA, and reconstructible involvement of the CHA without extension to the CA. Borderline PDA was defined by more than 180° contact to the SMA or the CA or thrombosis and involvement of the SMV or PV with no possibility of surgical reconstruction. Oral consent was obtained from all participants and an explicative notice was sent with the possibility to readdress it in case of refusal. The study 2021/640 was approved by our research department.

### CT scan protocol

All CT scans were performed with a 128 MD CT scanner (General Electric Revolution CT, General Electric). In routine practice, the CT scan protocol for PDA included non-contrast and multiphase contrast-enhanced acquisitions in the pancreatic arterial phase (acquired with bolus tracking, with a region of interest (ROI) located in the aorta, 20 s after a trigger of 120HU (absolute value)) and portal venous phase (80 s after contrast administration). Contrast administration was performed by intravenous injection of 1.5mL/kg non-ionic contrast medium at 400 mg I/mL through a consistent 30s injection (power injector at a rate of 3–5 mL/s). The CT scan was performed in the radiology department and imported into our treatment planning system (Varian Medical System) to create a fictional RT plan.

### Radiation therapy

Each patient had two CT scans, one acquired at the arterial phase and one at the venous phase. The GTV was delineated on the arterial and venous phases by two senior radiation oncologists (operators) specialized in gastro-intestinal cancers with more than five years of practice (8-year experience for operator 1 and 5-year experience for operator 2). All observers had full access to the clinical data and were blinded to each other during the process of delineation. The GTV delineated by a radiologist specialized in pancreatic imaging was the gold standard. The GTV included the visible pancreatic tumor. The clinical target volume (CTV) included the GTV without additional margin (CTV = GTV). The planning target volume (PTV) was created by adding a 1 cm margin in all directions to the CTV. The following organs at risk (OAR): stomach, duodenum, liver, bowel (including small bowel and colon), kidneys, and spinal cord, were delineated on the arterial and venous phases on one CT scan for each patient. Six fictional dosimetric plans were generated for each patient (one plan for each CT phase, for the radiologist and the two radiation oncologists, thus three arterial based plans and three venous based plans) to deliver a dose of 54 Gy in 30 fractions of 1.8 Gy, five fractions per week, using intensity-modulated RT (IMRT). For the OARs’ dose constraints, the ESTRO 2021 recommendations were applied (Additional file 1) [14].

### Geometric analysis of the delineated volumes

#### Inter-observer variability

The GTVs of the gold standard (GS-GTV) were compared to the operators’ GTVs at the arterial and the venous phases. The volume ratio (VR = GTV operator 1/GTV operator 2) between operator 1 and operator 2 was calculated with VR = 1 being optimal. For inter-observer variability, the Jaccard Conformity index (JCI), Geographical Miss Index (GMI), and kappa index were used (Additional file 2). JCI represents the ratio of the intersection of two volumes to the union of the two volumes (Additional file 2 A). A ratio of 1 represents a perfect conformity between the operators’ and the GS, while a ratio of 0 represents a complete mismatch. GMI is the ratio of the volume of the GS missed by the operators to the volume of the GS (Additional file 2B). A ratio of 1 represents a complete missed volume and 0 represents no miss. Thus, JCI indicates the degree of concordance between volumes and GMI indicates the percentage of missed tumor. Kappa index measures the inter-observer agreement (Additional file 2 C). This index was calculated with the GS to evaluate the variability among the three observers between the arterial and the venous phases. A ratio of 1 represents a perfect agreement, while 0 represents no agreement.

### Intra-observer variability

To evaluate the reproducibility in GTV volume delineation, the same operator redrew a GTV on the CT phases without referring to previous contours, at least one month after the first delineation. The second GTV volumes were then compared with the GTV volumes previously created. For these volumes, the percentage differences, referred to as delta operator, were calculated on the basis of the formula used by Cattaneo *and al.* defined in Additional file 2D [15]. This formula indicates the intra-observers’ volume variability combining both volume and position analysis.

### Statistical analysis

Patients’ characteristics were described with median and range for continuous variables, and with frequency and percentage for categorical variables. JCI, Kappa, and GMI were described with mean, standard deviation (SD), median, and range. For each patient, the mean values of GTV, PTV, JCI from operators measures and Kappa index were computed. Continuous variables (volumes and indices) were compared between arterial and venous phases on one hand, and between gold standard and operators on the other hand, with Wilcoxon signed rank test for paired samples. The significance level was set to *p* < 0.05 and should be interpreted according to the fact this was an exploratory study. To determine possible effects of the GTV volumes on the delineation concordance and accuracy, a linear regression model was fitted to individual JCI, GMI and kappa indices. The slope of each regression line β, R², and p values were calculated.

## Results

### Patients’ characteristics

Patients’ characteristics are shown in Additional file 3. The majority of the patients were active smokers. Abdominal pain was the main symptom leading to the diagnosis. Five patients underwent biliary stent placement due to obstructive icterus. PDA was localized in the pancreatic head in 60% of the patients. There were 60% of T2 tumors, 35% resectable, 45% borderline, and 20% locally advanced tumors. Twelve patients received FOLFIRINOX and two patients received FOLFOX, with a median number of 6 cycles (range, 2–12). Among these patients, ten went on to surgery and two to receive CRT. Six patients had no previous treatment, three had resectable tumors and ulteriorly were operated on, and the others ulteriorly received CT.

### Inter-observer variability

**Fig. 1:**
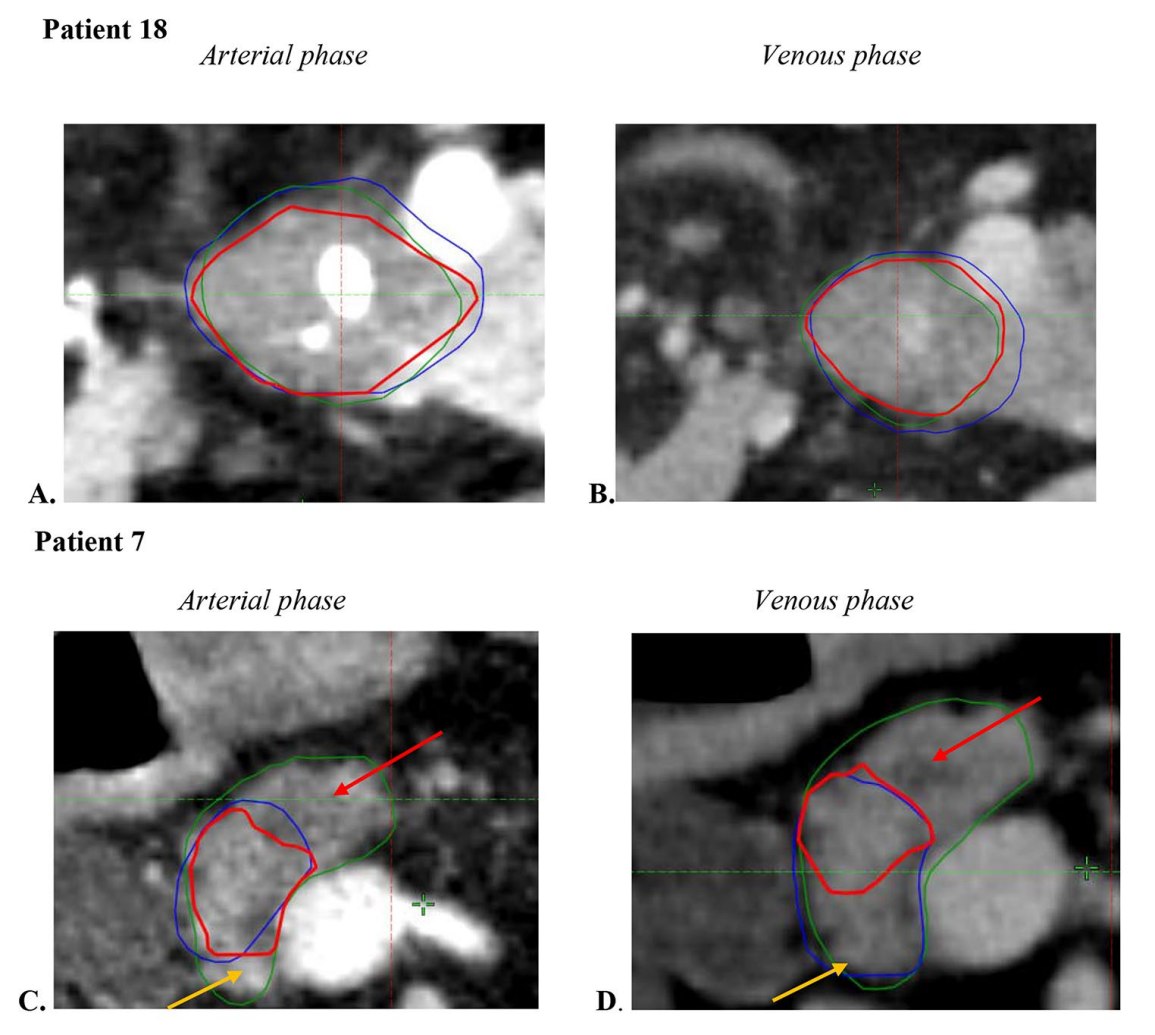
Representative axial CT scans showing inter-observer variability during delineation of the GTV in two patients. Patient 18 (**A**, **B**) represents the best JCI case (hypodense tumor with good concordance). Patient 7 (**C**, **D**) represents the worst JCI case (red arrow: iso-attenuating tumor with dilated bile duct; yellow arrow: normal pancreactic parenchyma) gsGTVs are shown in red; radiation oncologists’ GTVs are shown in blue and green at the arterial (**A**, **C**) and the venous phases (**B**, **D**)

**Fig. 2:**
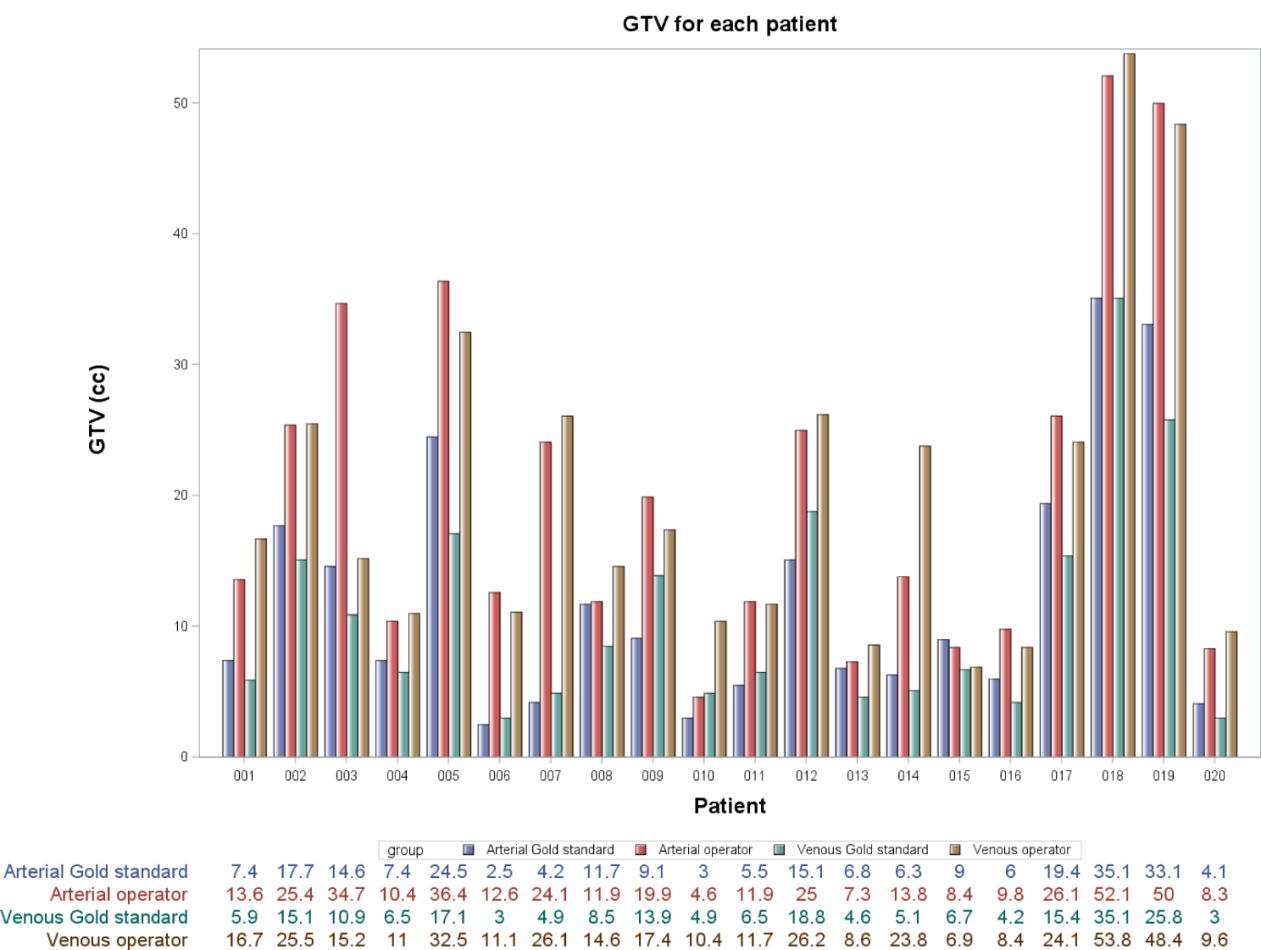
Bar diagram showing GTV for each patient averaged across the three observers

Two representative cases of GTV delineation by all three observers is shown in Fig. 1. The mean VR was 0.92 (+/- 0.47) at the arterial phase and 0.98 (+/- 0.48) at the venous phase for the operators. The median volume of the GS-GTVs were 8.2 cc (range, 2.5–35.1) at the arterial phase, and 6.6 cc (range, 3-35.1) at the venous phase (*p* = 0.056) (Table 1). For operator 1, median GTV was 12.85 cc on the arterial phase and 14.75 cc on the venous phase. For operator 2, median GTV was 17.10 cc and 16.85 cc, respectively. The median volume of the GTVs of the operators were 13.7 cc (range, 4.6–52.1) and 16.0 cc (range, 6.9–53.8) at the arterial and venous phases, respectively (*p* = 0.83). The GS-GTVs were smaller than the GTVs’ operators in all patients except for three patients (#10; #13; and #15) at the arterial phase, and one patient (#15) at the venous phase (Fig. 2). Compared to the operators’ median GTVs, the median GS-GTVs were significantly lower at the arterial phase (*p* < 0.0001) and the venous phase (*p* < 0.001) (Table 1).

**Table 1:** GTV and PTV volumes for all patients

	Gold standard			Operators (mean volume of the two radiation oncologists)			*P* value gold standard vs. operators	
	Arterial	Venous	*p* value	Arterial	Venous	*p* value	Arterial	Venous
GTV (cc), median (range)	8.2 (2.5–35.1)	6.6 (3-35.1)	0.056	13.7 (4.6–52.1)	16.0 (6.9–53.8)	0.83	< 0.0001	< 0.0001
PTV (cc), median (range)	64.4 (46-92.8)	51.4 (31.3-135.2)	0.019	81.0 (43.6-179.1)	84.5 (51.7-176.2)	0.47	< 0.0001	< 0.0001

The median JCI for the arterial and the venous phases were 0.5 (range, 0.17–0.64) and 0.41 (range, 0.23–0.61), respectively (*p* = 0.10) (Table 2). The median GMI were 0.07 (range, 0-0.79) and 0.05 (range, 0-0.39), respectively (*p* = 0.15) (Table 2). In only one case (patient 10), the GMI was ≥ 0.5 which corresponds to at least 50% of the tumor being missed (Additional file 4). There was a moderate agreement between the operators and the GS with a median kappa index of 0.52 (range, 0.38–0.57) in the arterial phase and 0.52 (range, 0.36–0.57) in the venous phase (*p* = 0.08) (Table 2).

**Table 2:** Mean and median values of the inter-observer variability indices

		Arterial phase	Venous phase	*P* value
JCI	mean (SD)	0.48 (0.14)	0.41 (0.14)	
	median (range)	0.5 (0.17–0.64)	0.41(0.23–0.61)	0.10
GMI	mean (SD)	0.12 (0.18)	0.09 (0.1)	
	median (range)	0.07 (0-0.79)	0.05 (0-0.39)	0.15
Kappa index	mean (SD) median (range)	0.5 (0.06) 0.52 (0.38–0.57)	0.48 (0.07) 0.52 (0.36–0.57)	0.08

**Fig. 3:**
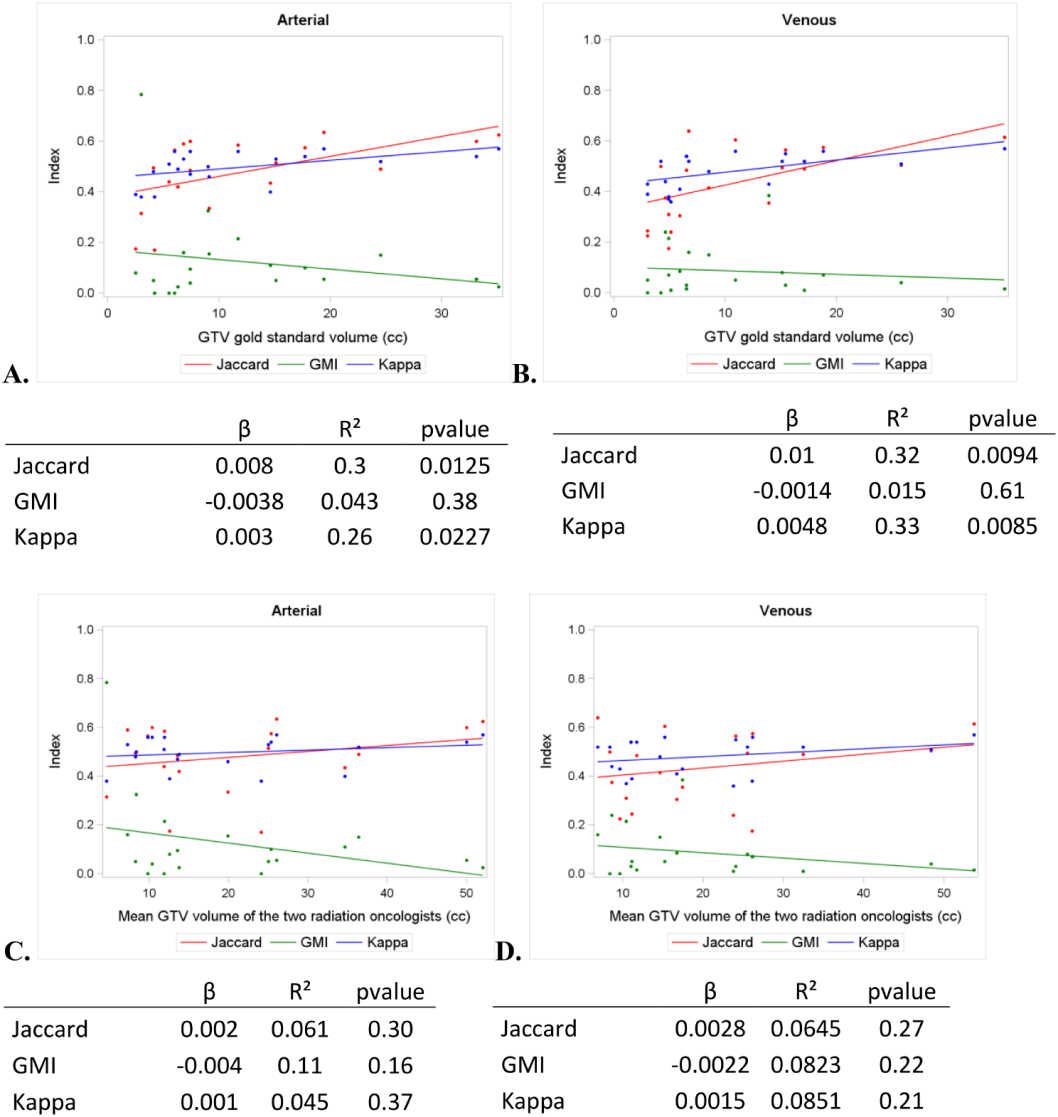
Scatter plot of the mean JCI, GMI and kappa index. Scatter plot according to gsGTV at arterial (**A**) and venous (**B**) phases and according to GTV of the two radiation oncologists at arterial (**C**) and venous (**D**) phases with linear regression model

Figure 3 shows the regression lines fitted to the indices at the arterial and venous phases for the GS and operators. At the arterial phase, a statistically significant association was found between JCI and kappa index (β = 0.008 (p value = 0.0125) and β = 0.003 (p value = 0.0227), respectively) and GS-GTVs but not with the operators’ GTVs. A high variability around the fitted line was found (R² =0.30 for JCI and 0.26 kappa index). Similar results were observed in venous phase. For GMI, no statistically significant association with GTV volume was found.

### Intra-observer variability

**Fig. 4:**
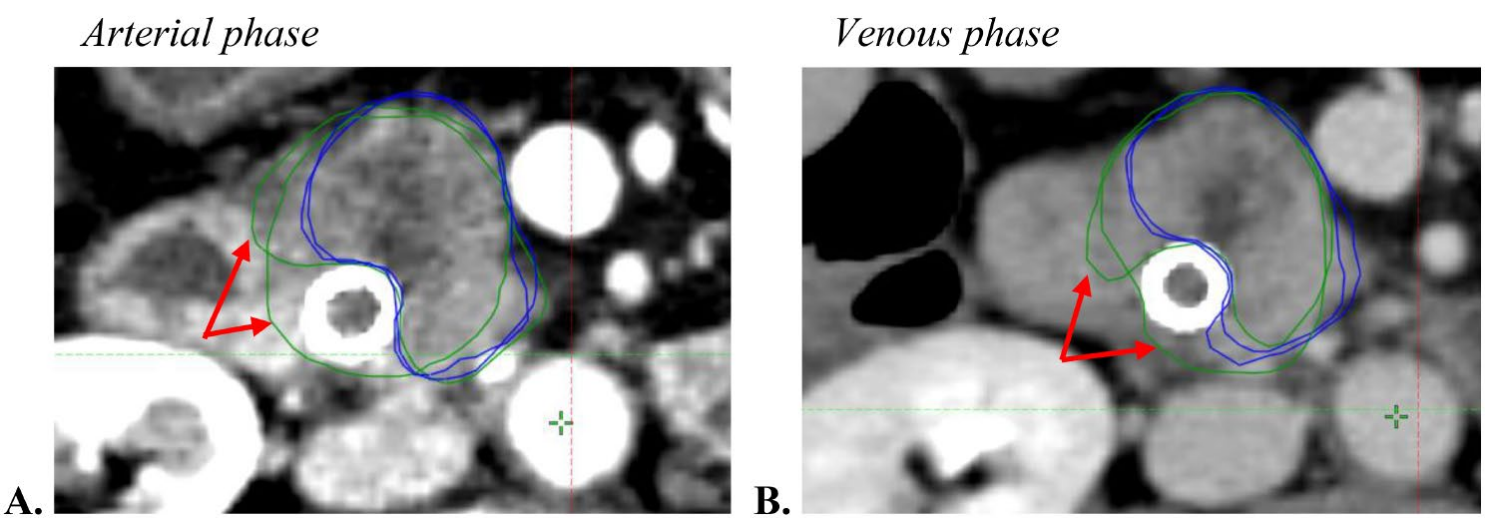
Representative axial CT scans showing intra-observer variability during delineation of the GTV in patient 8. Radiation oncologists’ GTVs are shown in blue and green at the arterial (**A**) and the venous phases (**B**). For patient 8 with an intermediate JCI, there was a variability in the delineation of the biliary stent (see red arrow) for the operator in green

The intra-observer variability is illustrated in Fig. 4. The intra-observer variability for GTV delineation was lower at the venous phase than at the arterial phase for the two operators (Table 3A). The median JCI was 0.61 (range, 0-58-0.62) at the arterial phase and 0.72 (range, 0.61–0.73) at the venous phase (Table 3B). The median delta operator was 39.8% (range 37.8–42) for the arterial phase and 27.9% (range 27.9–38.8) for the venous phase.

**Table 3A:** Intra-observer variability. Difference in GTV volumes between operator 1 (Op1) and operator 2 (Op2) for each CT phase

	GTV’s volume relative difference (%)			
ID	Arterial		Venous	
	Op 1	Op 2	Op 1	Op 2
004	34.6	51.6	15	15
007	-75.4	-31.7	-42.5	-25.7
008	0	-20.3	12.8	2.0

**Table 3B:** Intra-observer variability. Delta operator

	**Delta operator (%)** **(mean of the two operators)**			
**ID**	**Arterial**		**Venous**	
004	37.8		27.9	
007	42.1		38.8	
008	39.8		27.9	
For the three patients, median (range)	39.8 (37.8–42.1)		27.9 (27.9–38.8)	

### Impact on dose-volume histogram

There were no statistically significant differences between the two phases for the GS or the operators (Table 4). At the arterial phase, there were significant differences between GS and the operators for the stomach maximum dose and V45, duodenum V50 and V45, bowel V15, kidney mean dose, liver mean dose, and kidney V20.

**Table 4:** Dose-volume reporting for organs at risk at each CT phase for the gold standard and the operators (*n* = 20 patients)

		Gold standard			Operators			*P* value gold standard vs. operators	
		Arterial	Venous	*P* value	Arterial	Venous	*P* value	Arterial	Venous
**Dmax (Gy)**									
Duodenum	median (range)	54.3 (21.9–54.9)	54.3 (36.8–54.8)	0.71	54.4 (29.8–55.5)	54.3 (48-55.5)	0.79	0.085	0.1
	≤ 55 Gy	20 (100%)	20 (100%)		18 (90%)	17 (85%)			
	> 55 Gy	0 (0%)	0 (0%)		2 (10%)	3 (15%)			
Stomach	median (range)	51.9 (1-55.5)	51.6 (0.8–54.7)	0.6	50 (1.45-55)	50.3 (1.4–54.7)	0.45	0.03	0.07
	≤ 55 Gy	19 (95%)	20 (100%)		18 (90%)	20 (100%)			
	> 55 Gy	1 (5%)	0 (0%)		2 (10%)	0 (0%)			
Bowel	median (range)	39.4 (13.3–54.1)	40.4 (11.8–54.3)	0.33	45.8 (11.9–56)	43.5 (12.7–55.4)	1	0.07	0.02
	≤ 55 Gy	20 (100%)	20 (100%)		19 (95%)	19 (95%)			
	> 55 Gy	0 (0%)	0 (0%)		1 (5%)	1 (5%)			
**V50 Gy (%)**									
Duodenum	median (range)	4.35 (0–22)	2.9 (0.3–10.9)	0.38	10 (0-29.5)	9.7 (0-31.2)	0.1	0.006	0.0003
	≤ 10%	14 (70%)	14 (70%)		10 (50%)	10 (50%)			
	> 10%	6 (30%)	6 (30%)		10 (50%)	10 (50%)			
Stomach	median (range)	0 (0-4.9)	0 (0-4.1)	0.26	0.1 (0-7.25)	0.1 (0–6)	0.96	0.04	0.04
	≤ 10%	20 (100%)	20 (100%)		20 (100%)	20 (100%)			
	> 10%	0 (0%)	0 (0%)		0 (0%)	0 (0%)			
Bowel	median	0	0	1	0	0	1	0.06	0.06
	≤ 10%	20 (100%)	20 (100%)		20 (100%)	20 (100%)			
	> 10%	0 (0%)	0 (0%)		0 (0%)	0 (0%)			
**V50 Gy (cc)**									
Duodenum	median (range)	3.5 (0-24.7)	2.8 (0.3–10.4)	0.8	9 (0.5–31.5)	8.8 (0.5–17.3)	0.7	0.028	0.0008
	≤ 10 cc	17 (70%)	15 (75%)		9 (45%)	8 (40%)			
	> 10 cc	6 (30%)	5 (25%)		11 (55%)	12 (60%)			
Bowel	median (range)	0 (0-8.8)	0 (0-4.9)	0.8	0 (0–16)	0.02(0-10.2)	0.36	0.008	0.008
	≤ 10 cc	20 (100%)	20 (100%)		19 (95%)	19 (95%)			
	> 10 cc	0 (0%)	0 (0%)		0 (5%)	1 (5%)			
**V45 Gy (%)**									
Duodenum	median (range)	6.7 (0.1–27.4)	4.15 (0.4–13.9)	0.21	11.28 (0-39.9)	11.8 (0-54.2)	0.16	0.005	< 0.0001
	≤ 15%	15 (75%)	16 (80%)		10 (50%)	11 (55%)			
	> 15%	5 (25%)	4 (20%)		10 (50%)	9 (45%)			
Stomach	median (range)	0.2 (0-6.8)	0 (0-5.6)	0.26	0.35 (0-9.9)	0.25 (0-7.9)	0.84	0.02	0.008
	≤ 15%	20 (100%)	20 (100%)		20 (100%)	20 (100%)			
	> 15%	0 (0%)	0 (0%)		0 (0%)	0 (0%)			
Bowel	median (range)	0 (0–2)	0 (0-1.3)	0.75	0 (0-3.3)	0 (0-2.3)	0.1	0.008	0.03
	≤ 15%	20 (100%)	20 (100%)		20 (100%)	20 (100%)			
	> 15%	0 (0%)	0 (0%)		0 (0%)	0 (0%)			
**V45 Gy (cc)**									
Stomach	median (range)	0.45 (0-12.7)	0 (0-3.2)	0.12	1 (0-17.5)	0.78 (0-13.9)	0.83	0.06	0.002
	≤ 75 cc	20 (100%)	20 (100%)		20 (100%)	20 (100%)			
	> 75 cc	0 (0%)	0 (0%)		0 (0%)	0 (0%)			
**V15 Gy (cc)**									
Bowel	median (range)	54.1 (0-181.7)	53.2 (0-252.6)	0.87	75.7 (0-336.3)	86.5 (0-361)	0.9	0.001	0.0001
	≤ 120 cc	17 (85%)	17 (85%)		14 (70%)	14 (70%)			
	> 120 cc	3 (15%)	3 (15%)		6 (30%)	6 (30%)			
**Dmean(Gy)**									
Kidneys	median (range)	4.4 (0.6–11.3)	4.1 (0.1–10.9)	0.71	5.1 (0.85–12.7)	6 (1.1–12.9)	0.07	0.006	< 0.001
	≤ 18 Gy	20 (100%)	20 (100%)		20 (100%)	20 (100%)			
	> 18 Gy	0 (0%)	0 (0%)		0 (0%)	0 (0%)			
Liver	median (range)	2.4 (1-8.5)	2.1 (1-8.5)	0.0006	3.4 (1.3–8.7)	3.4 (1.1–7.8)	0.11	0.0011	< 0.0001
	≤ 25 GY	20 (100%)	20 (100%)		20 (100%)	20 (100%)			
	> 25 Gy	0 (0%)	0 (0%)		0 (0%)	0 (0%)			
**V20 (%)**									
Kidneys	median (range)	0 (0-10.9)	0.1 (0–11)	0.1	1 (0-7.7)	1 (0-10.5)	0.72	0.03	0.07
	≤ 32%	20 (100%)	20 (100%)		20 (100%)	20 (100%)			
	> 32%	0 (0%)	0 (0%)		0 (0%)	0 (0%)			
D0.1 cc (Gy)									
Spinal cord	median (range)	13.2 (6.9–22.1)	13.2 (3.9–24)	0.5	14.5 (5-22.9)	13.8 (6–23)	0.7	0.09	0.04
	≤ 45 Gy	20 (100%)	20 (100%)		20 (100%)	20 (100%)			
	> 44 Gy	0 (0%)	0 (0%)		0 (0%)	0 (0%)			

There were also some differences between the GS and the radiation oncologists in the venous phase planification for the bowels maximum dose and V15, duodenum V50 and V45, spinal cord D0.01 cc, stomach V50 and V45, kidney mean dose, liver mean dose, and spinal cord D0.01 cc.

## Discussion

In this study, we aimed at evaluating the inter-observer variability in the GTV delineation according to the injection phase between radiation oncologists and a radiologist specialized in PC. Since the arterial phase leads to enhanced contrast between the pancreatic gland and the tumor, we expected a lower variability at this phase. However, our results showed no difference between the arterial phase and the venous phase with respect to the JCI, GMI, and kappa index. Moreover, there was a poor agreement in the GTV delineation between the radiation oncologists and the radiologist. This was further highlighted by the significantly smaller GS-GTVs compared to the operators’ GTVs, which could also explain the differences seen in the dosimetric results. GMI was extremely low with no tumors completely missed. Although we failed to demonstrate a correlation between GTV on one hand, and JCI, GMI, and kappa index on the other hand, low volumes seemed to tend towards lack of agreement and geographical miss, suggesting difficulties in defining GTV for small tumors. In the SCALOP trial, it was shown that fewer tumors were missed when the GTV increased in size [8]. The trends observed with GTV volume can be explained by the dependence between the metrics and the volumes. JCI and GMI are known to be sensitive to structure size, especially small volumes. Thus, small delineation variations can have a large impact on these metrics. However, we believe that other factors can explain these results such as post-chemotherapy fibrosis, artefacts, and contrast differences between the tumor periphery, which is less hypodense than the tumor center, and the surrounding tissue where the contrast difference tends to diminish. Moreover, surrounding pancreatic parenchyma may be the site of pancreatic atrophy or chronic pancreatitis which can make the delineation difficult. In addition, some PC are discovered in an emergency context leading to the use of a metallic biliary stents. In this study, five patients had a biliary stent. The JCI for patient 9 was low, as already reported in other studies for patients with biliary stents [16, 17]. The inclusion or not of the biliary stent in the GTV can create inter-observer and intra-observer variations as shown in Fig. 4. There is no guideline regarding the necessity to include or exclude the biliary stent in the GTV in the literature. Also, there was no recommendation given in prospective protocols such as the PREOPANC trial. Finally, the stents may affect the dose distribution and lead to hot spots [18].

In this study, we evaluated the intra-physician variability in delineation with the Δv% formula also used by Cattaneo et al. [15]. In their study, one physician contoured GTV on all phases of a 4D-CT and drew GTV one month later to evaluate 4D-contouring reproducibility. They found 25% difference in the GTV volume for a chosen single phase of the 4D-CT. In our study, three tumors were delineated in both phases, one month apart, in a blinded mode and we found more variations (Δv = 40% at arterial phase and 31% at venous phase). However, these values were the average of two operators and not only one as the former study. We found moderate agreement for each operator with a median JCI of 0.61 on arterial phase and 0.72 on venous phases. Overall, our results suggested a lower intra-observer variability at the venous phase. Several factors of intra-observer variability were previously described, including the thickness of the slices, the difficulty in delimiting the cranio-caudal limits of certain structures, the poor image quality, and the low contrast of the soft tissues [19–21]. However, differences between two volumes performed by the same operator cannot be explained by these factors alone as they clearly reflect a difference in interpretation of the CT images.

Inter- and intra-observer variability in the delineation of target volumes and OAR is a source of uncertainty in radiation therapy. There might be several approaches to help reduce these variabilities, such as delineation standardized guidelines and online contouring workshops. It is well known that pancreatic tumors are difficult to distinguish from normal pancreas tissue on diagnostic CT scans. Therefore, exploitation of other imaging modalities, such as magnetic resonance imaging (MRI) or PET-CT may be a step forward to reduce the variation in delineation of pancreatic tumors. Indeed, other studies have shown that additional imaging may be helpful in the delineation of pancreatic tumors. For instance, the availability of MRI images during target delineation resulted in smaller target volumes and reduced the inter-observer variability in most of the delineated structures (17). The Radiosurgery and Stereotactic Radiotherapy Working Group of the German Society of Radiation Oncology (DEGRO) evaluated the influence of imaging modalities on the definition of the target volumes of locally advanced PC. Delineation was based on either a planning 4D CT with or without IV contrast, with or without PET/CT, and with or without diagnostic MRI. When comparing the imaging modalities for delineation, the best agreement for the GTV was achieved using PET/CT, and for the ITV and PTV using 4D PET/CT, in treatment position with abdominal compression [22]. Furthermore, a multidisciplinary collaboration could improve delineation not only with the radiologist/nuclearist, but also with the gastroenterologist. For instance, endoscopic ultrasound-guided fiducial markers are used as landmarks for image-guided radiotherapy (IGRT). These markers could also aid the definition of GTV if placed around the tumor, in a minimally invasive fashion.

Our study has several limitations. First, this was a small exploratory study on 20 patients, which limits the generalization of the results. Second, respiration-related tumor mobility was not considered. Pancreatic tumors are mobile and studies reported an average of 2 cm displacement in the craniocaudal direction [23, 24]. Contrast enhanced 4D-CT can be performed to create an internal target volume (ITV) with good image quality and pancreatic tumor enhancement [25, 26]. However the 4D-CT can sometimes overestimate or underestimate the respiratory-induced motion due to irregular breathing of patients [27]. It would be interesting to repeat this study in the future with a 4DCT ITV based approach. A technique of diaphragmatic compression, breath-holding or respiratory gating can also be used to reduce the tumor’s displacement. For centers treating on MRI-LINAC, the real-time MRI image allows a live tracking of the tumor. However, this approach is limited due to its high cost. Third, the gold standard volumes were those of a specialized radiologist in gastro-intestinal cancers, delineating for the first time. The GS-GTV were lower than the operators’ GTV probably due to a more premature stop of the delineation in extreme positions than the radiation oncologists. On the other hand, the tumors may have been overestimated by the operators or a microscopic extension may also have been included. A duo consisting of a radiation oncologist and a radiologist experienced in PC would probably be more relevant, emphasizing the need for an interdisciplinary collaboration. Of note, there was a trend towards larger GTV volumes for the radiologist and smaller GTV volumes for the operators on the arterial phase, although the difference between arterial and venous phases was not statistically significant. GTV volume was slightly higher on the arterial phase for operator 2, which is similar to what was observed with the radiologist. Even though both operators had > 5-year experience, operator 1 had 8-year experience while operator 2 had 5-year experience, which could have introduced a bias. Finally, metrics measuring the overlap between structures, such as JCI, are not designed to uncover differences in distances or shapes. The Hausdorff distance (HD) would have been useful to describe such differences.

## Conclusion

This study provides a first approach of the impact of the arterial versus the venous phase enhancement in RT planning for PC. Our results suggest the combination of the two phases should be recommended and emphasize the need to provide training for radiation oncologists in pancreatic imaging and to collaborate within a multisciplinary team.

### Electronic supplementary material

Below is the link to the electronic supplementary material.

Supplementary Material 1

Supplementary Material 2

Supplementary Material 3

Supplementary Material 4

## Data Availability

The datasets used and/or analyzed during the current study are available from the corresponding author on reasonable request.

## Abbreviations

CA: Coeliac axis
CHA: Common hepatic artery
CT: Chemotherapy
CRT: Chemoradiation
CT scan: Computed tomography scan
CTV: Clinical target volume
GMI: Geographical Miss Index
GS: Gold standard
GTV: Gross tumour volume
GS-GTV: GTVs of the gold standard
IMRT: Intensity-modulated RT
ITV: Internal target volume
JCI: Jaccard conformity index
MRI: Magnetic resonance imaging
OAR: Organs at risk (OAR)
PC: Pancreatic cancer
PDA: Pancreatic ductal adenocarcinoma
PTV: Planning target volume
PV: Portal vein
RT: Radiation therapy
SBRT: Stereotactic body RT
SD: Standard deviation
SMA: Superior mesenteric artery
SMV: Superior mesenteric vein
