# Supplementary material for: A comparison of target volumes drawn on arterial and venous phase scans during radiation therapy planning for patients with pancreatic cancer: the PANCRINJ study
Source: Radiat Oncol. 2024 Jul 15;19:90. doi: 10.1186/s13014-024-02477-8 (PMC11251351; doi:10.1186/s13014-024-02477-8)
Supplement: Supplementary file 1 — Supplementary Material 1 [file 13014_2024_2477_MOESM1_ESM.docx]

**Additional File 1. Dose constraints for long-course CRT (1.8-2 Gy/fraction) from ESTRO ACROP guidelines 2020** (14)


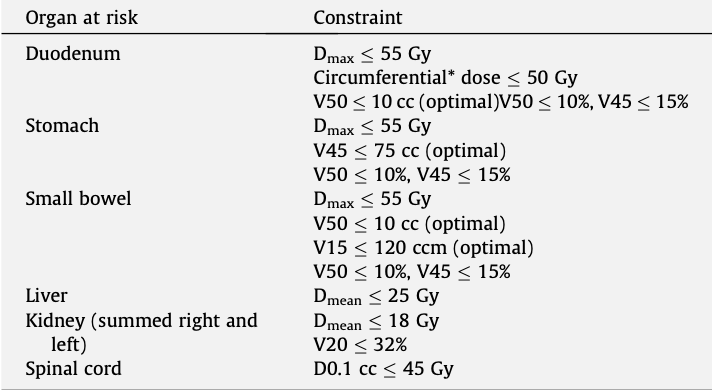


* Circumferential dose means that the dose not only at the adjacent side of the hollow organ wall next to the PTV but also at the opposite aspect of the wall, i.e. the dose is applied to the entire circumference of a segment of the hollow organ.
