# Supplementary material for: A comparison of target volumes drawn on arterial and venous phase scans during radiation therapy planning for patients with pancreatic cancer: the PANCRINJ study
Source: Radiat Oncol. 2024 Jul 15;19:90. doi: 10.1186/s13014-024-02477-8 (PMC11251351; doi:10.1186/s13014-024-02477-8)
Supplement: Supplementary file 2 — Supplementary Material 2 [file 13014_2024_2477_MOESM2_ESM.docx]

**Additional File 2. Inter and intra-observer variability indices.**

1. Jaccard conformity index (JCI)


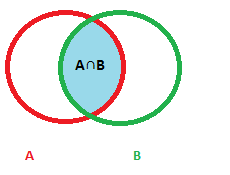

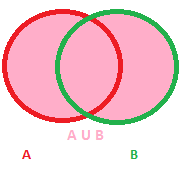

$$JCI= \frac{A \cap B}{A \cup B}$$

1. Geographical Miss Index (GMI)  C) Kappa index


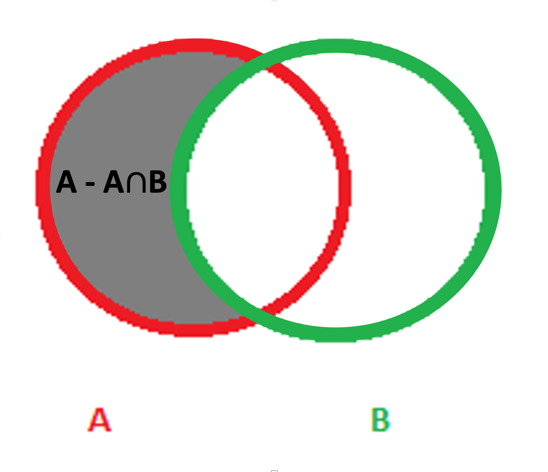

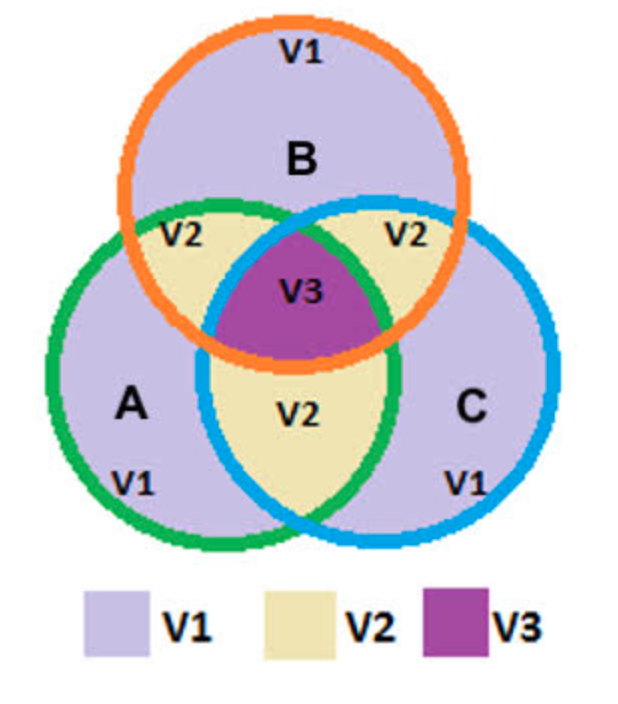


$GMI= \frac{A-(A\cap B)}{A}$ Kappa index = $\frac{V2+3V3}{V1+2V2+3V3}$

A = volume of the gold standard, B = volume operator 1, C = volume of operator 2, V1 = volume delineated by one operator, V2 = common volume delineated by two operators, V3 = common volume delineated by all three observers, A ∩ B = volume of intersection of A and B, A ∪ B = volume of union of A and B.

1. **Intra-observer variability**

$$\Delta v=100* \frac{(A \cup B- A\cap B)}{A\cup B}$$

$\Delta v$ ; delta volume, percentage differences between the two volumes of the same operator. A = first volume of one operator, B = second volume of the same operator, A ∩ B = volume of intersection of A and B, A ∪ B = volume of union of A and B.
