# Supplementary material for: A comparison of target volumes drawn on arterial and venous phase scans during radiation therapy planning for patients with pancreatic cancer: the PANCRINJ study
Source: Radiat Oncol. 2024 Jul 15;19:90. doi: 10.1186/s13014-024-02477-8 (PMC11251351; doi:10.1186/s13014-024-02477-8)
Supplement: Supplementary file 3 — Supplementary Material 3 [file 13014_2024_2477_MOESM3_ESM.docx]

**Additional File 3. Patients’ characteristics**

|  |  | N=20  n (%) |  |
| --- | --- | --- | --- |
| **Age**, median (range) |  | 64.5 (52-83) |  |
| **WHO performance status,** median  0  1 |  | 6 (30)  14 (70) | |
| **Sex**  Men |  | 10 (50) | |
| Women |  | 10 (50) | |
| **Smoker**  Yes |  | 12 (60) | |
| **Pain at diagnosis**  Yes |  | 15 (75) | |
| **Icterus at diagnosis**  No  Yes |  | 17 (70)  6 (30) | |
| **If yes, biliary stent**  Yes |  | 5 (83) | |
| **Histology**  Adenocarcinoma |  | 20 (100) | |
| **Tumor vascular contact**  No  Yes |  | 4 (20)  16 (80) | |
| **If yes, n=16** |  |  |  |
| **SMV / PV**  Contact <180% without vein contour irregularity  Contact ≥ 180°  Contact < 180° with vein contour irregularity but no thrombosis  Contact <180 with thrombosis with safe vein reconstruction  Thrombosis with unsafe vein reconstruction |  | 6 (37.5)  5 (31.3)  3 (18.8)  1 (6.3)  1 (6.3) | |
|  |  |  | |
| **SMA**  No contact  Contact <180°  Contact ≥ 180° |  | 10 (62.5)  3 (18.8)  3 (18.8) | |
|  |  |  | |
| **CHA**  No contact  Contact without extension to CA or hepatic artery bifurcation with safe reconstruction  Unsafe artery reconstruction |  | 11 (68.8)  3 (18.8  2 (12.5) | |
| **CA**  No contact  Contact <180 |  | 14 (87.5)  2 (12.5) | |
| **Pancreatic tumor**  Resectable  Borderline  Locally advanced tumor |  | 7 (35)  9 (45)  4 (20) | |
| **T stage**  T1  T2  T3  T4 |  | 1 (5)  12 (60)  4 (20)  3 (15) | |
| **N stage**  N0  N1 |  | 16 (80)  4 (20) | |
| **M stage**  M0 |  | 20 (100) | |
| **Stage**  IA  IB  IIA  IIB  III |  | 1 (5)  9 (45)  4 (20)  3 (15)  3 (15) | |
| **Localization**  Head  Isthmus  Body  Tail |  | 12 (60)  4 (20)  2 (10)  2 (10) | |
| **Previous treatment**  No  Yes |  | 6 (30)  14 (70) | |
| **If yes, n=14** |  |  | |
| CRT  No  RT  No  CT  FOLFIRINOX  FOLFOX  **Number of cycles of CT, median (range)**  **Treatment after CT**  CT |  | 14 (100)  14 (100)  12 (85.7)  2 (14.3)  6 (2-12)  2 (14) | |
| Surgery  CRT |  | 10 (71.4)  2 (14.3) | |
| **If no previous treatment, n=6, intention**  Surgery  CT |  | 3 (50)  3 (50) | |

Abbreviations: WHO, world health organization; mm, millimeter; SMV, Superior mesenteric vein; PV, portal vein; SMA, superior mesenteric artery ; CHA, common hepatic artery ; CA, celiac axis; CRT, chemoradiation ; CT, chemotherapy ; RT, radiation therapy
