# Supplementary material for: A comparison of target volumes drawn on arterial and venous phase scans during radiation therapy planning for patients with pancreatic cancer: the PANCRINJ study
Source: Radiat Oncol. 2024 Jul 15;19:90. doi: 10.1186/s13014-024-02477-8 (PMC11251351; doi:10.1186/s13014-024-02477-8)
Supplement: Supplementary file 4 — Supplementary Material 4 [file 13014_2024_2477_MOESM4_ESM.docx]

**Additional File 4.** **Mean and median values of the indices**

A) Individual

|  | JCI (mean of the two radiation oncologists) | | GMI (mean of the two radiation oncologists) | | Kappa index | |
| --- | --- | --- | --- | --- | --- | --- |
| ID | arterial | venous | arterial | venous | arterial | venous |
| 001 | 0.49 | 0.31 | 0.1 | 0.09 | 0.47 | 0.41 |
| 002 | 0.58 | 0.5 | 0.1 | 0.08 | 0.54 | 0.52 |
| 003 | 0.44 | 0.61 | 0.11 | 0.05 | 0.4 | 0.56 |
| 004 | 0.6 | 0.54 | 0.04 | 0.03 | 0.56 | 0.54 |
| 005 | 0.49 | 0.49 | 0.15 | 0.01 | 0.52 | 0.52 |
| 006 | 0.18 | 0.25 | 0.08 | 0.05 | 0.39 | 0.39 |
| 007 | 0.17 | 0.18 | 0 | 0.07 | 0.38 | 0.38 |
| 008 | 0.59 | 0.41 | 0.22 | 0.15 | 0.56 | 0.48 |
| 009 | 0.34 | 0.36 | 0.16 | 0.39 | 0.46 | 0.43 |
| 010 | 0.32 | 0.31 | 0.79 | 0.22 | 0.38 | 0.37 |
| 011 | 0.44 | 0.485 | 0 | 0.015 | 0.51 | 0.54 |
| 012 | 0.52 | 0.575 | 0.05 | 0.07 | 0.53 | 0.56 |
| 013 | 0.59 | 0.375 | 0.16 | 0.24 | 0.53 | 0.44 |
| 014 | 0.42 | 0.24 | 0.025 | 0.01 | 0.49 | 0.36 |
| 015 | 0.5 | 0.64 | 0.33 | 0.16 | 0.5 | 0.52 |
| 016 | 0.57 | 0.5 | 0 | 0 | 0.56 | 0.52 |
| 017 | 0.64 | 0.57 | 0.055 | 0.03 | 0.57 | 0.55 |
| 018 | 0.63 | 0.62 | 0.025 | 0.015 | 0.57 | 0.57 |
| 019 | 0.6 | 0.51 | 0.055 | 0.04 | 0.54 | 0.51 |
| 020 | 0.5 | 0.23 | 0.05 | 0 | 0.48 | 0.43 |
